# KCNQ Currents and Their Contribution to Resting Membrane Potential and the Excitability of Interstitial Cells of Cajal From the Guinea Pig Bladder

**DOI:** 10.1016/j.juro.2009.02.108

**Published:** 2009-07

**Authors:** Ursula A. Anderson, Christopher Carson, Karen D. McCloskey

**Affiliations:** Division of Basic Medical Sciences (Physiology), School of Medicine and Dentistry, Queen's University, Belfast, United Kingdom

**Keywords:** urinary bladder, KCNQ potassium channels, membrane potentials, electrophysiology, muscle, smooth, ATP, adenosine triphosphate, BK, large conductance Ca^2+^ activated K^+^ channel, ICC, interstitial cells of Cajal, ICC-LP, lamina propria ICC, MFA, meclofenamic acid, SMC, smooth muscle cell, XE991, 10,10-bis(4-pyridinyl-methyl)-9(10H)-anthracenone

## Abstract

**Purpose:**

The presence of novel KCNQ currents was investigated in guinea pig bladder interstitial cells of Cajal and their contribution to the maintenance of the resting membrane potential was assessed.

**Materials and Methods:**

Enzymatically dispersed interstitial cells of Cajal were patch clamped with K^+^ filled pipettes in voltage clamp and current clamp modes. Pharmacological modulators of KCNQ channels were tested on membrane currents and the resting membrane potential.

**Results:**

Cells were stepped from −60 to 40 mV to evoke voltage dependent currents using a modified K^+^ pipette solution containing ethylene glycol tetraacetic acid (5 mM) and adenosine triphosphate (3 mM) to eliminate large conductance Ca activated K channel and K_adenosine triphosphate_ currents. Application of the KCNQ blockers XE991, linopirdine (Tocris Bioscience, Ellisville, Missouri) and chromanol 293B (Sigma®) decreased the outward current in concentration dependent fashion. The current-voltage relationship of XE991 sensitive current revealed a voltage dependent, outwardly rectifying current that activated positive to −60 mV and showed little inactivation. The KCNQ openers flupirtine and meclofenamic acid (Sigma) increased outward currents across the voltage range. In current clamp mode XE991 or chromanol 293B decreased interstitial cell of Cajal resting membrane potential and elicited the firing of spontaneous transient depolarizations in otherwise quiescent cells. Flupirtine or meclofenamic acid hyperpolarized interstitial cells of Cajal and inhibited any spontaneous electrical activity.

**Conclusions:**

This study provides electrophysiological evidence that bladder interstitial cells of Cajal have KCNQ currents with a role in the regulation of interstitial cell of Cajal resting membrane potential and excitability. These novel findings provide key information on the ion channels present in bladder interstitial cells of Cajal and they may indicate relevant targets for the development of new therapies for bladder instability.

The discovery of ICC in the bladder has provided the opportunity to investigate a novel potential control mechanism of bladder smooth muscle activity. Several subtypes of ICC are present in the bladder wall, including ICC-LP, intramuscular ICC on the boundary of detrusor smooth muscle bundles and interbundle ICC between smooth muscle bundles. There are reported differences in the morphological and physiological properties of ICC-LP and the detrusor ICC subpopulations. For example, ICC-LP form a network of stellate-shaped cells connected by gap junctions that respond to purinergic agonists but do not respond to cholinergic stimulation.^1^ However, detrusor ICC are spindle-shaped cells with lateral branches that do not form networks, but rather respond to cholinergic agonists by firing Ca^2+^ transients.^2^, ^3^ ICC-LP have been proposed to participate in sensory processing, whereas intramuscular ICC have been suggested to modulate the activity of neighboring smooth muscle.

K^+^ channels have an important ubiquitous role in regulating resting membrane potential and, therefore, the excitability of many cell types. Guinea pig detrusor ICC have several types of K^+^ channels, including BK and voltage dependent delayed rectifiers.^4^ A component of total K^+^ current in detrusor ICC was not identified by McCloskey,^4^ which may be attributable to less well-known voltage dependent K^+^ channels such as KCNQ (Kv7). There is reason to believe that KCNQ currents might be present in bladder cells because Streng et al noted decreased capsaicin induced bladder overactivity or hypercontractility when the KCNQ channel opener retigabine was delivered intravesically in conscious rats.^5^

KCNQs are voltage dependent K^+^ channels that have been widely studied in the nervous system, heart and inner ear.^6^ Mutations in these channels lead to cardiac arrhythmias, such as long QT syndrome and sudden death,^7^ benign familial neonatal convulsions or epilepsy^8^ and autosomal dominant progressive deafness.^9^ To date 5 gene members of the KCNQ family have been identified, each encoding a different KCNQ α-subunit.^1^, ^2^, ^3^, ^4^, ^5^ Considerable diversity in KCNQ expression has been noted among tissues with channels expressed as homomultimers or heteromultimers with accessory subunits, such as the KCNE β-subunits.

We determined whether a component of the outward K^+^ current in guinea pig bladder ICC was due to KCNQ. Also, we assessed any contribution of KCNQ channels to the regulation of ICC resting membrane potential.

## Materials and Methods

### Cell Preparation and Electrophysiological Recordings

The bladder was removed from adult guinea pigs of either sex weighing 250 to 600 gm. The animals had been sacrificed by cervical dislocation or injection with pentobarbitone in accordance with Schedule 1 United Kingdom Home Office regulations. The bladders were opened and the mucosa was removed to leave the underlying detrusor. Cells were enzymatically isolated from the detrusor region, as previously described.^2^

Cells were plated to the bottom of a recording bath and constantly perfused with Hanks' physiological salt solution at room temperature. The cell of interest was continuously superfused by a drug delivery system consisting of a pipette with a tip diameter of 200 μm placed approximately 300 μm away. The solution in the drug delivery system could be switched to one containing a drug with a dead space time of less than 10 seconds. ICC were identified by the presence of lateral branches as previously described^2^, ^4^ and selected for patch clamp recordings using the whole cell or the amphotericin perforated patch technique. Patch pipette electrodes made from borosilicate glass (resistance 2 to 4 MΩ) were connected via an analog to digital/digital to analog converter (National Instruments™) to an Axopatch 1D patch clamp amplifier (Axon Instruments, Foster City, California) and a personal computer running WinWCP software (http://spider.science.strath.ac.uk/sipbs/software_ses.htm). In whole cell mode after gigaseals were obtained access to the cell interior was gained by sharp suction. In perforated patch recordings the cell was given a test depolarization from −60 to 40 mV every 30 seconds until the outward current developed to its maximal amplitude. Series resistance and the capacitive surge were compensated using circuitry from the Axopatch 1D amplifier.

### Drugs and Solutions

Hanks' physiological salt solution was composed of 130 mM Na^+^, 5.8 mM K^+^, 135 mM Cl^−^, 4.16 mM HCO_3_^−^, 0.44 mM H_2_PO_4_^−^, 0.34 mM HPO_4_^2−^, 0.4 mM SO_4_^2−^, 1.8 mM Ca^2+^, 0.9 mM Mg^2+^, 10 mM HEPES, 10 mM glucose and 2.9 mM sucrose pH buffered to 7.40 with NaOH. Modified pipette solution to eliminate the contribution from BK and K_ATP_ was composed of 130 mM K^+^, 6.2 mM Na^+^, 110 mM gluconate, 21 mM Cl^–^, 0.5 mM Mg^2+^, 3 mM ATP (Na^+^ salt), 0.1 mM guanosine triphosphate, 5 mM HEPES and 5 mM EGTA adjusted to pH 7.2 with KOH. Stock solutions of XE991, linopirdine and MFA were dissolved in distilled water. Chromanol 293B and flupirtine were dissolved in dimethyl sulfoxide.

Data were analyzed with WinWCP, Microsoft® Excel® and GraphPad Prism® (Graphpad). Statistical comparisons were made using Student's t test with p <0.05 level considered significant. Summary data are expressed as the mean ± SEM.

## Results

### KCNQ Blockers and ICC Outward Currents

McCloskey identified a BK and voltage dependent delayed rectifier K^+^ current in ICC and described a noninactivating, voltage dependent unidentified component.^4^ The possibility that novel KCNQ currents might contribute to the unidentified portion of ICC outward current was explored by testing the effect of KCNQ pharmacological drugs. Detrusor ICC were voltage clamped using K^+^ filled pipettes containing 5 mM EGTA and 3 mM ATP to inhibit BK and K_ATP_ channels, respectively, and depolarized to evoke outward currents. This approach was successfully used by Yeung and Greenwood.^10^ It avoids the use of channel blocking drugs, which may interfere with the conductance of interest. Outward currents were dominant, although inward Ca^2+^ currents were also present. Mean ICC capacitance was 59 ± 1 pF in 80 cells.

**Figure 1:**
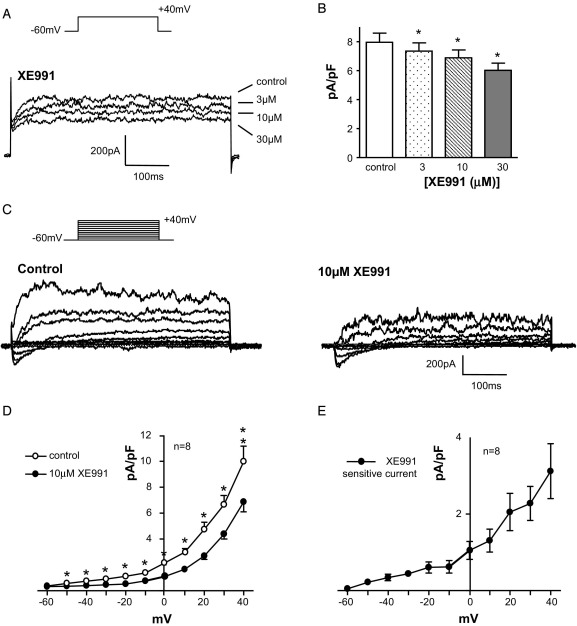
XE991 effect on ICC outward currents. *A*, ICCs were stepped from −60 to 40 mV to evoke outward currents. K^+^ pipette solution contained 5 mM EGTA and 3 mM ATP to eliminate BK and K_ATP_ currents. XE991 application dose dependently decreased outward current. *ms*, milliseconds. *B*, there was significant decrease by XE991 in 11 cells. Asterisk indicates p <0.05. *C*, current family elicited by stepping from −60 to 40 mV in 10 mV increments. Currents were decreased by 10 μM XE991. *D*, Current-voltage plot demonstrates relationship between mean current and test potentials in absence and presence of XE991. *E*, KCNQ current was obtained by subtracting XE991 insensitive current from control current, activated positive to −60 mV. It was outwardly rectifying.

A depolarizing step from −60 to 40 mV elicited noninactivating currents (fig. 1,  *A*). The KCNQ blocker XE991^11^ decreased the currents in a concentration dependent manner. Figure 1, *B* shows summary data on 11 cells. XE991 (10 μM) significantly decreased the outward current from 498 ± 44 to 432 ± 39 pA and 30 μM further decreased the current to 376 ± 34 pA (each p <0.05). The current-voltage relationship was examined by stepping from −60 mV to a range of potentials up to 40 mV in 10 mV increments. The resulting family of currents was decreased by 10 μM XE991 across the voltage range (fig. 1, *C*). Mean current at each potential was plotted against the test potential in the absence and presence of drug (fig. 1, *D*). Subtracting XE991 sensitive currents from control currents revealed that the XE991 sensitive KCNQ current was a voltage dependent, outwardly rectifying current that activated positive to −60 mV (fig. 1, *E*).

**Figure 2:**
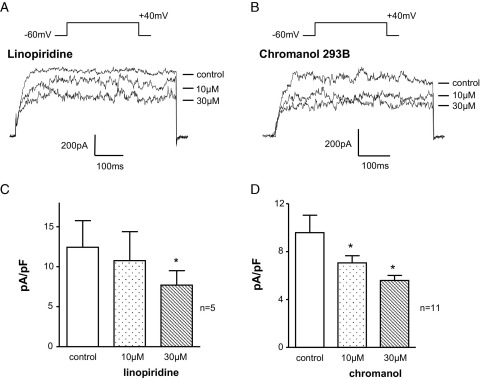
Effect of other KCNQ blockers on ICC outward current. *A*, ICCs were stepped from −60 to 40 mV. Control current was decreased by 10 and 30 μM linopirdine. *ms*, milliseconds. *B*, outward currents were also sensitive to 10 and 30 μM chromanol 293B. *C*, effect of linopirdine in 5 cells. Asterisk indicates p <0.05. *C* and *D*, effect of chromanol in 11 cells.

Another 2 drugs that are often used to block KCNQ channels were tested. Figure 2, *A* shows the concentration dependent decrease in current amplitude (evoked by stepping from −60 to 40 mV) by the lesser potent analogue of XE991, linopirdine.^12^, ^13^ Although it was less effective than XE991, linopirdine (10 μM) decreased control current from 689 ± 130 to 585 ± 155 pA in 5 cells, while increasing the concentration to 30 μM further decreased the current to 435 ± 94 pA (p >0.05). Chromanol 293B has been used as a specific KCNQ1 channel blocker.^14^ Chromanol (10 μM) significantly decreased the control current of 490 ± 70 to 368 ± 39 pA and 30 μM further decreased the residual current to 292 ± 29 pA in 11 cells each (each p <0.05, fig. 2, *B* and *D*).

### KCNQ Openers and ICC Outward Currents

**Figure 3:**
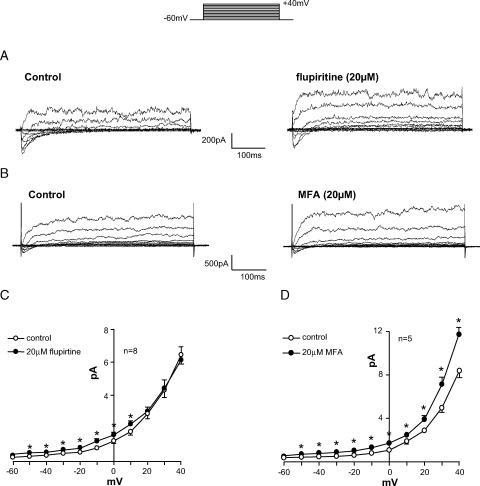
KCNQ opener effect on ICC outward current. *A*, current family evoked by stepping from −60 to 40 mV in 10 mV increments. Flupirtine application increased outward current amplitude. *ms*, milliseconds. *B*, control currents in different cell and MFA effect on outward currents. *C*, flupirtine effect in 8 cells. *D*, summary data on MFA effect in 5 cells.

The effect of the KCNQ channel openers flupirtine and MFA were then tested on ICC outward currents (fig. 3). Flupirtine acts as an anticonvulsant and muscle relaxant but it was more recently shown to activate KCNQ currents, resulting in decreased excitability in neurons.^15^ Flupirtine increased the family of outward currents, typical in 8 experiments (fig. 3, *A*). Likewise MFA, a well-known anti-inflammatory drug and a KCNQ 2/3 K^+^ channel opener in neurons,^16^ also increased outward currents in 5 ICC (fig. 3, *B*).

### KCNQ Drugs and ICC Resting Membrane Potential

**Figure 4:**
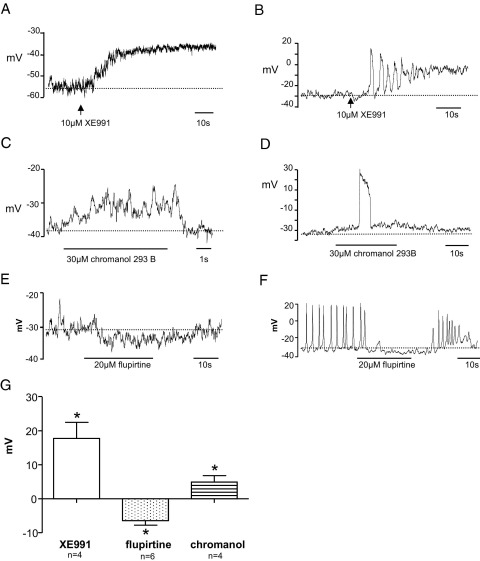
KCNQ pharmacological agents and resting membrane potential. *A*, ICCs were current clamped to record resting membrane potential. XE991 application depolarized cell. *s*, seconds. *B*, in previously quiescent cell XE991 induced firing of spontaneous transient depolarizations, followed by sustained depolarization. *C*, chromanol 293B application also depolarized cell. *D*, depolarization by chromanol induced spontaneous transient depolarization firing. *E*, flupirtine application increased resting membrane potential. *F*, hyperpolarization by flupirtine inhibited spontaneous electrical activity in active cell. *G*, effect of KCNQ drugs on membrane potential. Asterisk indicates p <0.05.

Cells were patched with amphotericin B filled pipettes using the modified pipette solution and studied in current clamp mode, so that the effect of KCNQ drugs on resting membrane potential could be assessed. Mean resting membrane potential in 18 ICC under these conditions was −38 ± 3 mV. While most recordings were electrically quiescent, spontaneous oscillations in resting membrane potential were observed under control conditions in 5 of 18 cells. Application of XE991 (10 μM) depolarized the membrane potential and mean depolarization in 4 cells was 18 ± 5 mV (p <0.05, fig. 4, *A*). XE991 evoked transient depolarizations before sustained depolarization in 1 of 4 cells (fig. 4, *B*). Chromanol 293B also depolarized 4 ICC by 5 ± 2 mV (p <0.05), although its effect was less marked than that of XE991 (fig. 4, *C*). Chromanol evoked electrical activity in 3 of 4 cells (fig. 4, *D*). The KCNQ opener flupirtine produced a mean membrane hyperpolarization of 6 ± 1 mV in 6 cells (p <0.001, fig. 4, *E*), of which 2 were spontaneously active. Flupirtine reversibly abolished spontaneous activity in these 2 cells (fig. 4, *F*). Figure 4, *G* shows the overall effect of KCNQ channel drugs on ICC resting membrane potential.

## Discussion

KCNQ currents have classically been investigated in neuronal and cardiac cells and tissues. More recently KCNQ currents have been reported to be present in smooth muscle preparations, including the pulmonary artery,^17^ portal vein^18^ and mesenteric artery,^19^ and to have a role in smooth muscle excitability and vascular tone regulation. The current study shows that, in addition to neuronal, cardiac and smooth muscle cells, ICC from guinea pig detrusor also have KCNQ currents. We recently characterized similar currents in detrusor smooth muscle, in which KCNQ currents appear to have a key role in the control of bladder spontaneous electrical and contractile activity (Anderson and McCloskey, unpublished data).

In the current study KCNQ currents from bladder ICC showed many typical properties of KCNQ channels, including voltage dependence, activation positive to −60 mV, outward rectification and little inactivation. They were sensitive to inhibition by drugs commonly used to block KCNQ channels, ie XE991, linopirdine and chromanol 293B. XE991 and linopirdine have been reported to be effective KCNQ blockers, although the block was not found to be specific to a particular KCNQ channel subtype.^12^, ^13^ XE991 was more potent for decreasing the residual outward current in ICC compared with linopirdine in the current study, consistent with the findings of others.^13^ A decrease in currents by XE991 and linopirdine similar to those in the current study were found in vascular smooth muscle cells from rat mesenteric arteries^19^ and the rat portal vein.^10^

Further support for the existence of KCNQ currents in bladder ICC comes from experiments with the KCNQ channel openers flupirtine and MFA, which each increased outward currents, consistent with findings in rat mesenteric artery smooth muscle cells.^19^ Flupirtine and its more potent analogue retigabine increased KCNQ/M current and decreased excitability in neurons.^15^, ^16^ Moreover, MFA was found to augment KCNQ currents by shifting the voltage activation curve to the left and slowing deactivation kinetics, leading to membrane hyperpolarization.^16^

The potential physiological significance of KCNQ currents in bladder ICC was demonstrated in current clamp studies of cell resting membrane potential. Activation of KCNQ currents in bladder ICC appears to decrease excitability since application of the channel opener flupirtine hyperpolarized ICC and inhibited the firing of spontaneous transient depolarizations. This implies that KCNQ channel opening could act as a brake, preventing electrical activity and depolarization of the cells. Consistent with this the blockade of KCNQ channels depolarized the membrane potential sufficiently to trigger firing in otherwise quiescent cells.

The precise roles of ICC in the bladder have not been fully elucidated. However, several studies indicate that they act to modify or regulate the activity of neighboring SMCs.^3^, ^20^ If this is indeed the case, blockade of KCNQ channels on ICC would depolarize the ICC and subsequently affect SMCs via gap junctions or evoke Ca^2+^ release from internal stores in ICC and, therefore, activate cellular signaling mechanisms between ICC and SMCs. Conversely KCNQ openers would hyperpolarize ICC, which would then hyperpolarize neighboring SMCs. Therefore, ICC may act to amplify the effect of KCNQ drugs on SMCs, representing a synergy of design. For example, SMC hyperpolarization by KCNQ opening while neighboring ICC remained depolarized would be unlikely to be maintained because the more positive ICC would tend to depolarize the SMCs. Thus, the presence of KCNQ channels on ICC as well as SMCs might represent a fine tuning mechanism to control bladder contractility. Streng et al reported compelling evidence for the functional role of KCNQ channels in the bladder in in vivo experiments in rats.^5^ In their study intravesical administration of the KCNQ opener retigabine decreased baseline bladder pressures in the rat detrusor, increased micturition volume and decreased capsaicin induced detrusor overactivity. Our study supports these findings and provides electrophysiological evidence for the existence of KCNQ channels in bladder ICC. It seems feasible that the development of bladder specific KCNQ openers could provide treatment for overactive bladder. Moreover, gene mutations of KCNQ subunits, such as cardiovascular, nervous and auditory pathologies, may well underlie bladder disorders.

## Conclusions

We investigated the hypothesis that a component of the outward K^+^ current in guinea pig bladder ICC is mediated by KCNQ channels. ICC outward currents were decreased by KCNQ pharmacological blockers and enhanced by KCNQ channel openers. Data suggest that KCNQ currents have a role in the regulation of ICC resting membrane potential and excitability. These novel findings provide key information on the ion channels present in bladder ICC and they may indicate relevant targets for the development of new therapy for bladder instability.
